# Supplementary material for: Cross-sectional study on public health knowledge among first-year university students in Japan: Implications for educators and educational institutions
Source: PLoS One. 2023 Sep 8;18(9):e0291414. doi: 10.1371/journal.pone.0291414 (PMC10490915; doi:10.1371/journal.pone.0291414)
Supplement: S1 Table — (DOCX) [file pone.0291414.s001.docx]

***Supplemental table 1. List of specific knowledge questions regarding public health topics and answer choices (multiple answer)***

| ***Lifestyle-related diseases*** | | | |
| --- | --- | --- | --- |
|  | Q3 | Which lifestyle-related diseases do you know? | |
|  |  |  | Hypertension |
|  |  |  | Dyslipidemia |
|  |  |  | Type 2 diabetes |
|  |  |  | Chronic Kidney Disease (CKD) |
|  |  |  | Hyperuricemia/Gout |
|  |  |  | Obesity/Metabolic Syndrome |
|  |  |  | Fatty Liver/Non-alcoholic Fatty Liver Disease (NAFLD)/Non-alcoholic steatohepatitis (NASH) |
|  |  |  | Alcoholic hepatitis |
|  |  |  | Chronic obstructive pulmonary disease (COPD, emphysema and chronic bronchitis) |
|  |  |  | NONE |
|  |  |  | cancer |
|  |  |  | Cerebrovascular disease |
|  |  |  | IHD: Ischemic Heart Disease |
| ***Contraception*** | | |  |
|  | Q68 | Do you know the contraceptive methods below. | |
|  |  |  | Condoms |
|  |  |  | Rhythm method |
|  |  |  | Oral contraceptives |
|  |  |  | IUD (Inter Uterine Device: ) |
|  |  |  | IUS（Intra Uterine System) |
|  |  |  | Contraceptive surgery (males thread/cut vases, women thread/cut fallovules) |
|  |  |  | Other |
|  |  |  | abstinence(Other) |
|  |  |  | oral sex(Other) |
|  |  |  | circumcision(Other) |
| ***LGBTQIA+ stand for*** | | | |
|  | Q78 | Do you know what LGBTQIA+ represents? | |
|  |  |  | L : Lesbian |
|  |  |  | G : Gay |
|  |  |  | B : Bisexual |
|  |  |  | T :Transgender |
|  |  |  | Q : Queer |
|  |  |  | Q : Questioning |
|  |  |  | I : Intersex |
|  |  |  | A : Allies |
|  |  |  | A : Asexual |
|  |  |  | P : Pansexual |
|  |  |  | Didn't know any of them. |
| ***Pathogens that cause infections*** | | | |
|  | Q85 | Choose the pathogens you know that cause infections described below . | |
|  |  |  | Virus |
|  |  |  | Parasite |
|  |  |  | Fungus |
|  |  |  | Prion |
|  |  |  | Other |
|  |  |  | maternal infection |
|  |  |  | Oral infection |
|  |  |  | Aerosol infection |
| ***Transmission of infection*** | | | |
|  | Q88 | Choose what you know about the transmission described below. | |
|  |  |  | Contact transmission |
|  |  |  | Droplet transmission |
|  |  |  | Airborne transmission |
|  |  |  | vector-borne transmission |
|  |  |  | Vertical transmission |
|  |  |  | Other |
|  |  |  | maternal infection |
|  |  |  | Oral infection |
|  |  |  | Aerosol infection |
