# Supplementary material for: Cross-sectional study on public health knowledge among first-year university students in Japan: Implications for educators and educational institutions
Source: PLoS One. 2023 Sep 8;18(9):e0291414. doi: 10.1371/journal.pone.0291414 (PMC10490915; doi:10.1371/journal.pone.0291414)
Supplement: S2 Table — (DOCX) [file pone.0291414.s002.docx]

| ***Supplemental Table 2. Inventory of questionnaire items, along with individual question response rates and corresponding keywords in tables.*** | | | | |  |  |  |  |
| --- | --- | --- | --- | --- | --- | --- | --- | --- |
|  |  | ***Key words*** |  | ***Had knowledge/ Had no knowledge*** |  | ***Sources of information*** |  | ***Self-assessment of knowledge (1-10)*** |
| ***Nutrition and Physical Activity*** | | | (%) |  |  |  |  |  |
|  | Topic 1 | Lifestyle-related diseases | Q4  (99.6) | Do you know the general preventive measures for lifestyle-related diseases (e.g., moderate exercise, balanced eating habits, quitting smoking, etc.)? | Q5  (99.6) | What are the sources of information about lifestyle-related diseases? | Q6  (99.5) | How much do you think you know about lifestyle-related diseases? |
|  | Topic 2 | Food poisoning | Q7  (100) | Do you know that food poisoning could be caused by food like fugu or poisonous mushrooms, or viruses and bacteria in food? | Q8  (98.5) | What are the sources of information about food poisoning? | Q9  (100) | How much do you think you know about food poisoning? |
|  | Topic 3 | Allergic reaction | Q10  (99.5) | Do you know about the fact that certain foods and medications can cause allergic reactions and in some cases be life-threatening? | Q11  (99.3) | What are the sources of information about allergic reactions? | Q12  (99.5) | How much do you think you know about allergic reactions? |
| ***Alcohol, Tobacco, and Other Drugs*** | | |  |  |  |  |  |  |
|  | Topic 4 | Health effects of smoking | Q13  (100) | Do you know about the health effects of smoking? | Q14  (99.8) | What are the sources of information about the health effects of smoking? | Q15  (100) | How much do you think you know about the health effects of smoking? |
|  | Topic 5 | Health hazards of passive smoking | Q16  (99.6) | Do you know about the health hazards caused by passive smoking? | Q17  (99.5) | What are the sources of information about the health hazards caused by passive smoking? | Q18  (100) | How much do you think you know about the health hazards of passive smoking? |
|  | Topic 6 | Smoking effects on pregnancy | Q19 (100) | Do you know about the possibility that smoking can cause premature births and miscarriages in pregnant women? | Q20 (97.3) | What are the sources of information about the possibility of premature birth or miscarriage caused by smoking? | Q21 (99.6) | How much do you think you know about the effects of pregnant women smoking? |
|  | Topic 7 | Dependence of smoking | Q22 (99.8) | Do you know about the dependence caused by smoking? | Q23 (99.5) | What are the sources of information about the dependence on smoking? | Q24 (99.6) | How much do you think you know about the dependence on smoking? |
|  | Topic 8 | Health effects of e-cigarettes | Q25 (99.8) | Do you know that e-cigarettes (e-cigarettes) also have harmful effects on health? | Q26 (80.5) | What are the sources of information about the health effects of e-cigarettes (e-cigarettes)? | Q27 (99.6) | How much do you think you know about the health effects of e-cigarettes (e-cigarettes)? |
|  | Topic 9 | Effect of alcohol consumption | Q28 (99.8) | Do you know about the effects of alcohol consumption (suppressing brain function, slowing judgment and body movement, and acute alcohol poisoning)? | Q29 (99.1) | What are the sources of information about the effects of alcohol consumption (suppressing brain function and slowing judgment and body movements, or acute alcoholism)? | Q30 (99.8) | How much do you think you know about the effects of alcohol consumption? |
|  | Topic 10 | Alcohol consumption effect on pregnancy | Q31 (99.8) | Do you know about the effects on the fetus of pregnant women drinking alcohol (e.g., underweight or brain damage)? | Q32 (96.5) | What are the sources of information about the effects on the fetus of pregnant women drinking alcohol (underweight or brain damage)? | Q33 (99.3) | How much do you think you know about the effects on the fetus of pregnant women drinking alcohol? |
|  | Topic 11 | Mental/physical effect of mind-altering drugs | Q34 (99.6) | Do you know that designer drugs (illegal and unregulated drugs) along with cannabis, stimulants(e.g., methamphetamine), and MDMA (ecstasy) are dangerous drugs that affect the mind and body? | Q35 (99.8) | What are the sources of information about the mental and physical effects of cannabis, stimulants, MDMA, and designer drugs? | Q36 (99.8) | How much do you think you know about the mental and physical effects of illegal drugs such as cannabis, stimulants, MDMA, and designer drugs? |
|  | Topic 12 | Alcohol, smoking, and drug addiction | Q37 (100) | Do you know that dependence on alcohol, smoking, and drugs could be both mental and physical dependence/addiction? | Q38 (97.8) | What are the sources of information about alcohol, smoking, and drug dependence/addiction? | Q39 (100) | How much do you think you know about alcohol, smoking, and drug dependence/addiction? |
|  | Topic 13 | Addiction | Q40 (99.8) | Do you know that addiction is one type of mental illness? | Q41 (88.0) | What are the sources of information about addiction? | Q42 (98.5) | How much do you think you know about addiction? |
| ***Growth, Development, and Sexual Health*** | | |  |  |  |  |  |  |
|  | Topic 14 | Ovulation and menstruation | Q43 (100) | Do you know about the periodicity of ovulation and menstruation? | Q44 (99.1) | What are the sources of information about ovulation and menstruation? | Q45 (100) | How much do you think you know about ovulation and menstruation? |
|  | Topic 15 | Ejaculation | Q46 (99.6) | Do you know about ejaculation (the release of semen containing sperm out of the body due to mental and physical sexual excitement or stimulation)? | Q47 (98.2) | What are the sources of information about ejaculation? | Q48 (100) | How much do you think you know about ejaculation? |
|  | Topic 16 | How to use condoms | Q49 (99.6) | Do you know how to use condoms? | Q50 (87.1) | What are the sources of information about how to use condoms? | Q51 (98.9) | How much do you think you know about using condoms? |
|  | Topic 17 | Woman's basal body temperature | Q52 (99.6) | Do you know that there are various changes in a woman's basal body temperature (body temperature measured in a rested state that consumes only the minimum energy necessary for life support)? |  |  |  |  |
|  | Topic 18 | Measurement of woman's basal body temperature | Q53 (98.9) | Do you know how to measure a woman's basal body temperature (such as measuring with a female thermometer while lying quietly in bed when you wake up every morning)? | Q54 (77.0) | What are the sources of information about a woman's basal body temperature? | Q55 (98.9) | How much do you think you know about a woman's basal body temperature? |
|  | Topic 19 | Fertilization and pregnancy | Q56 (99.8) | Do you know that pregnancy is caused by sperm entering the egg and fertilized eggs being implanted in the endometrium? | Q57 (99.5) | What are the sources of information about fertilization and pregnancy (in general)? | Q58 (99.5) | How much do you think you know about fertilization and pregnancy (in general)? |
|  | Topic 20 | Gestation period and childbirth | Q59 (99.6) | Do you know that child is delivered through the birth canal after a gestation period of about 280 days (40 weeks) with the start of the last menstrual period as day 0? | Q60 (97.1) | What are the sources of information about pregnancy and childbirth (in general)? | Q61 (99.6) | How much do you think you know about pregnancy and childbirth (in general)? |
|  | Topic 21 | Public services related to pregnancy | Q62 (99.8) | Do you know about public services related to pregnancy (issuance of maternal and child health handbooks, parents' class, etc.)? | Q63 (91.6) | What are the sources of information about public services related to pregnancy (maternal and child health handbooks, parents' studies, etc.)? | Q64 (99.5) | How much do you think you know about public services related to pregnancy? |
|  | Topic 22 | Significance and importance of family planning | Q65 (99.8) | Do you know the significance and importance of making plans (appropriate family planning) based on social and economic conditions, conditions such as the child's growing environment, maternal health conditions, the timing, number, interval, etc. of pregnancies and childbirths? | Q66 (81.8) | What are the sources of information about the significance and importance of family planning? | Q67 (98.5) | How much do you think you know about the significance and importance of family planning? |
|  | Topic 23 | Contraception |  |  | Q69 (99.5) | What are the sources of information about contraception? | Q70 (99.3) | How much do you think you know about contraception? |
|  | Topic 24 | Abortion under Maternal Health Act in Japan | Q71 (99.5) | Do you know about abortion being allowed up to 21 weeks of pregnancy in Japan if there is a special reason under Maternal Health Act? | Q72 (90.7) | What are the sources of information about abortion under the Maternal Health Act in Japan? | Q73 (98.9) | How much do you think you know about abortion under Maternal Health Act in Japan? |
|  | Topic 25 | sex and gender | Q74 (99.8) | Do you know that sex is determined by biology whereas gender is a socially/culturally constructed expectation or identity? | Q75 (86.3) | What are the sources of information about sex and gender? | Q76 (99.1) | How much do you think you know about sex and gender? |
|  | Topic 26 | LGBTQIA+ | Q77 (99.5) | Do you know about LGBTQIA+ representing gender diversity, gender identity? | Q79 (90.0) | What are the sources of information about LGBTQIA+? | Q80 (98.7) | How much do you think you know about LGBTQIA+? |
| ***Personal and Community Health*** | | |  |  |  |  |  |  |
|  | Topic 27 | Birth control pills and preventing sexually transmitted diseases | Q81 (99.5) | Do you know that birth control pills are ineffective in preventing sexually transmitted diseases? |  |  |  |  |
|  | Topic 28 | Prevention of sexually transmitted diseases | Q82 (99.1) | Do you know that sexually transmitted diseases such as HIV could have no apparent symptoms and can spread infection? | Q83 (97.1) | What are the sources of information on how to prevent sexually transmitted diseases? | Q84 (99.3) | How much do you think you know about the prevention of sexually transmitted diseases? |
|  | Topic 29 | Pathogens that cause infections |  |  | Q86 (98.4) | What are the sources of information about pathogens that cause infections? | Q87 (98.9) | How much do you think you know about pathogens that cause infections? |
|  | Topic 30 | Transmission of infection |  |  | Q89 (98.9) | What are the sources of information about the transmission of infection? | Q90 (99.1) | How much do you think you know about the transmission of infection? |
|  | Topic 31 | Vaccines | Q91 (98.9) | Do you know that vaccination causes the body to produce antibodies to neutralize pathogens as well as to create a memory of resistance to infections? | Q92 (59.6) | What are the sources of information about vaccines? | Q93 (97.4) | How much do you think you know about vaccines? |
| ***Answer choices*** | |  |  |  |  |  |  |  |
|  |  |  | 1 | Learned it in class | 1 | Teacher(s) | 1 | No knowledge at all |
|  |  |  | 2 | Learned it outside of class | 2 | Parent(s) | 10 | Enough knowledge to teach someone else |
|  |  |  | 3 | Learned it both in class and outside of classes | 3 | Sibling(s) |  |  |
|  |  |  | 4 | Didn't learn it in class | 4 | Relative(s) |  |  |
|  |  |  | 5 | Had the class but don't remember | 5 | Friend(s) |  |  |
|  |  |  | 6 | Other sources | 6 | Textbook(s) |  |  |
|  |  |  |  |  | 7 | Books other than textbooks |  |  |
|  |  |  |  |  | 8 | Internet |  |  |
|  |  |  |  |  | 9 | Other |  |  |
