# Supplementary material for: Cross-sectional study on public health knowledge among first-year university students in Japan: Implications for educators and educational institutions
Source: PLoS One. 2023 Sep 8;18(9):e0291414. doi: 10.1371/journal.pone.0291414 (PMC10490915; doi:10.1371/journal.pone.0291414)
Supplement: S3 Table — (DOCX) [file pone.0291414.s003.docx]

***Supplemental Table 3 .Comparison of Students' previous knowledge between sex***

| Multiple answer |  | Sex | | | |
| --- | --- | --- | --- | --- | --- |
|  |  | Male(%) | Female(%) | Total(%) | *p* |
| Lifestyle-related diseases | |  |  |  |  |
|  | Hypertension | 169(88.02) | 327(92.63) | 498(90.71) | NS |
|  | Dyslipidemia | 51(26.56) | 112(31.73) | 164(29.87) | NS |
|  | Type 2 diabetes | 108(56.25) | 248(70.25) | 358(65.21) | .020 |
|  | Chronic Kidney Disease (CKD) | 21(10.94) | 48(13.6) | 70(12.75) | NS |
|  | Hyperuricemia/Gout | 63(32.81) | 136(38.53) | 200(36.43) | NS |
|  | Obesity/Metabolic Syndrome | 163(84.9) | 325(92.07) | 491(89.44) | NS |
|  | Fatty Liver/Non-alcoholic Fatty Liver Disease (NAFLD)/Non-alcoholic steatohepatitis (NASH) | 33(17.19) | 69(19.55) | 103(18.76) | NS |
|  | Alcoholic hepatitis | 82(42.71) | 184(52.12) | 268(48.82) | NS |
|  | Chronic obstructive pulmonary disease (COPD, emphysema and chronic bronchitis) | 27(14.06) | 65(18.41) | 93(16.94) | NS |
| Contraceptive methods | |  |  |  |  |
|  | Condoms | 192(100) | 344(97.45) | 540(98.36) | NS |
|  | Rhythm method | 95(49.48) | 169(47.88) | 267(48.63) | NS |
|  | Oral contraceptives | 172(89.58) | 324(91.78) | 500(91.07) | NS |
|  | IUD | 48(25) | 97(27.48) | 147(26.78) | NS |
|  | IUS | 12(6.25) | 48(13.6) | 62(11.29) | .024 |
|  | Contraceptive surgery | 89(46.35) | 165(46.74) | 256(46.63) | NS |
| Gender/Orientation definition | |  |  |  |  |
|  | L : Lesbian | 156(81.25) | 297(84.14) | 456(83.06) | NS |
|  | G : Gay | 154(80.21) | 303(85.84) | 461(83.97) | NS |
|  | B : Bisexual | 144(75) | 270(76.49) | 417(75.96) | NS |
|  | T :Transgender | 145(75.52) | 291(82.44) | 439(79.96) | NS |
|  | Q : Queer | 14(7.29) | 50(14.16) | 65(11.84) | NS |
|  | Q : Questioning | 28(14.58) | 75(21.25) | 104(18.94) | NS |
|  | I : Intersex | 18(9.38) | 43(12.18) | 62(11.29) | NS |
|  | A : Allies | 3(1.56) | 13(3.68) | 16(2.91) | NS |
|  | A : Asexual | 23(11.98) | 64(18.13) | 89(16.21) | NS |
|  | P : Pansexual | 14(7.29) | 48(13.6) | 64(11.66) | NS |
|  | Didn't know any of them. | 25(13.02) | 33(9.35) | 58(10.56) | NS |
| Pathogens that cause infections | |  |  |  |  |
|  | Bacteria | 173(90.1) | 334(94.62) | 511(93.08) | NS |
|  | Virus | 169(88.02) | 331(93.77) | 504(91.8) | .024 |
|  | Parasite | 104(54.17) | 239(67.71) | 344(62.66) | .007 |
|  | Fungus | 23(11.98) | 62(17.56) | 85(15.48) | NS |
|  | Prion | 17(8.85) | 20(5.67) | 38(6.92) | NS |
| Transmission | |  |  |  |  |
|  | Contact transmission | 185(96.35) | 350(99.15) | 539(98.18) | .013 |
|  | Droplet transmission | 165(85.94) | 329(93.2) | 498(90.71) | .007 |
|  | Airborne transmission | 151(78.65) | 311(88.1) | 466(84.88) | .004 |
|  | vector-borne transmission | 66(34.38) | 136(38.53) | 204(37.16) | NS |
|  | Vertical transmission | 10(5.21) | 26(7.37) | 37(6.74) | NS |

NOTE. Percentages are parenthesis represent portion of valid N of each category. Results are based on two-sided tests. Tests are adjusted for all pairwise comparisons.
