# Supplementary material for: Cross-sectional study on public health knowledge among first-year university students in Japan: Implications for educators and educational institutions
Source: PLoS One. 2023 Sep 8;18(9):e0291414. doi: 10.1371/journal.pone.0291414 (PMC10490915; doi:10.1371/journal.pone.0291414)
Supplement: Attachment — Submitted filename: PONE-D-23-16475-R1.pdf [file pone.0291414.s005.pdf]

PLOS ONE

Cross-Sectional Study on Public Health Knowledge among First-Year University Students in Japan: Implications for Educators and Educational Institutions

--Manuscript Draft--

Manuscript Number:	PONE-D-23-16475
Article Type:	Research Article
Full Title:	Cross-Sectional Study on Public Health Knowledge among First-Year University Students in Japan: Implications for Educators and Educational Institutions
Short Title:	Public Health Knowledge among First-Year University Students in Japan
Corresponding Author:	Miwa Sekine, Ph.D<br>Juntendo University: Juntendo Daigaku<br>Tokyo, JAPAN
Keywords:	
Abstract:	In recent years, health knowledge gaps and/or biases in health knowledge have become increasingly prevalent. These phenomena have become particularly noticeable and problematic during the COVID-19 pandemic and have further widened the spread of misinformation and knowledge disparities. With constant exposure to disinformation and misinformation through television, the internet, and social media, the lack of accurate public health knowledge is present even among healthcare-related university students. This study aimed to examine university students' knowledge on basic public health topics prior to admission to assess whether they had adequate knowledge before learning about further specialized education. Participants in this cross-sectional study were first-year students from medical schools, health-related colleges, and liberal arts colleges. A self-administered electronic survey was conducted from April to May 2021 at a private university in Japan, comprising six colleges with seven programs. Data analysis was conducted from June to December 2022 using students' self-reported public health knowledge, sources of information, and self-assessment of knowledge levels. Among the 1,562 students who received the questionnaire, 549 [192 male students (35%), 353 female students (64.3%), and 4 undisclosed (0.7%)] responded to at least one question. The results revealed that health knowledge, particularly sexual health knowledge, was limited, and at least 10% of students reported not learning in class before university admission the following 11 topics: two on Alcohol, Tobacco, and Other Drugs; eight on Growth, Development, and Sexual Health; and one on Personal and Community Health. These results indicated that there are significant knowledge gaps and biases as well as gender gaps concerning public health, especially sexual health, which may serve educators and educational institutions to better understand and prepare for further specialized education and suggest a need to supplement and reinforce the foundation of public health knowledge in healthcare majors at the time of university admission.
Order of Authors:	Miwa Sekine, Ph.D
	David Aune
	Shuko Nojiri
	Makino Watanabe
	Yuko Nakanishi
	Shinobu Sakurai
	Tomomi Iwashimizu
	Yasuaki Sakano
	Tetsuya Takahashi
	Yuji Nishizaki
Additional Information:	

Powered by Editorial Manager® and ProduXion Manager® from Aries Systems Corporation

Question	Response
**Financial Disclosure**<br><br>Enter a financial disclosure statement that describes the sources of funding for the work included in this submission. Review the submission guidelines for detailed requirements. View published research articles from *PLOS ONE* for specific examples.<br><br>This statement is required for submission and **will appear in the published article** if the submission is accepted. Please make sure it is accurate.<br><br>**Unfunded studies**<br>Enter: *The author(s) received no specific funding for this work.*<br><br>**Funded studies**<br>Enter a statement with the following details:<br>• Initials of the authors who received each award<br>• Grant numbers awarded to each author<br>• The full name of each funder<br>• URL of each funder website<br>• Did the sponsors or funders play any role in the study design, data collection and analysis, decision to publish, or preparation of the manuscript?<br>• **NO** - Include this sentence at the end of your statement: *The funders had no role in study design, data collection and analysis, decision to publish, or preparation of the manuscript.*<br>• **YES** - Specify the role(s) played.<br><br>* typeset	Initials of the authors who received each award: MS<br>Grant number: 18K13259<br>The full name of funder: JSPS KAKENHI<br>URL of funder website: https://www.jsps.go.jp/english/e-grants/<br><br>The funders had no role in study design, data collection and analysis, decision to publish, or preparation of the manuscript.
**Competing Interests**<br><br>Use the instructions below to enter a competing interest statement for this submission. On behalf of all authors, disclose any competing interests that could be perceived to bias this work—acknowledging all financial support and any other relevant financial or non-financial competing interests.	The authors have declared that no competing interests exist.

This statement is required for submission and **will appear in the published article** if the submission is accepted. Please make sure it is accurate and that any funding sources listed in your Funding Information later in the submission form are also declared in your Financial Disclosure statement.<br><br>View published research articles from *PLOS ONE* for specific examples.<br><br>**NO authors have competing interests**<br><br>Enter: *The authors have declared that no competing interests exist.*<br><br>**Authors with competing interests**<br><br>Enter competing interest details beginning with this statement:<br><br>*I have read the journal's policy and the authors of this manuscript have the following competing interests: [insert competing interests here]*<br><br>* typeset	
**Ethics Statement**<br><br>Enter an ethics statement for this submission. This statement is required if the study involved:<br><br>• Human participants<br>• Human specimens or tissue<br>• Vertebrate animals or cephalopods<br>• Vertebrate embryos or tissues<br>• Field research<br><br>Write "N/A" if the submission does not require an ethics statement.<br><br>General guidance is provided below. Consult the submission guidelines for detailed instructions. **Make sure that all information entered here is included in the Methods section of the manuscript.**	This study was approved by the Juntendo University Ethical Review Board (No. 2020324). We obtained informed consent from respondents by asking to tick the checkbox in the survey and collected the completed questionnaires. Respondents who did not provide informed consent were excluded from the study. Parental consent was not required since first-year students in Japan are 18 years of age or older. There were no other exclusion criteria.

Powered by Editorial Manager® and ProduXion Manager® from Aries Systems Corporation

**Format for specific study types**<br><br>**Human Subject Research (involving human participants and/or tissue)**<br>• Give the name of the institutional review board or ethics committee that approved the study<br>• Include the approval number and/or a statement indicating approval of this research<br>• Indicate the form of consent obtained (written/oral) or the reason that consent was not obtained (e.g. the data were analyzed anonymously)<br><br>**Animal Research (involving vertebrate animals, embryos or tissues)**<br>• Provide the name of the Institutional Animal Care and Use Committee (IACUC) or other relevant ethics board that reviewed the study protocol, and indicate whether they approved this research or granted a formal waiver of ethical approval<br>• Include an approval number if one was obtained<br>• If the study involved *non-human primates*, add *additional details* about animal welfare and steps taken to ameliorate suffering<br>• If anesthesia, euthanasia, or any kind of animal sacrifice is part of the study, include briefly which substances and/or methods were applied<br><br>**Field Research**<br><br>Include the following details if this study involves the collection of plant, animal, or other materials from a natural setting:<br>• Field permit number<br>• Name of the institution or relevant body that granted permission	
**Data Availability**<br><br>Authors are required to make all data underlying the findings described fully available, without restriction, and from the time of publication. PLOS allows rare exceptions to address legal and ethical concerns. See the PLOS Data Policy and FAQ for detailed information.	Yes - all data are fully available without restriction

Powered by Editorial Manager® and ProduXion Manager® from Aries Systems Corporation

A Data Availability Statement describing where the data can be found is required at submission. Your answers to this question constitute the Data Availability Statement and **will be published in the article**, if accepted.<br><br>**Important:** Stating 'data available on request from the author' is not sufficient. If your data are only available upon request, select 'No' for the first question and explain your exceptional situation in the text box.<br><br>Do the authors confirm that all data underlying the findings described in their manuscript are fully available without restriction?	
**Describe where the data may be found in full sentences. If you are copying our sample text, replace any instances of XXX with the appropriate details.**<br><br>• If the data are **held or will be held in a public repository**, include URLs, accession numbers or DOIs. If this information will only be available after acceptance, indicate this by ticking the box below. For example: *All XXX files are available from the XXX database (accession number(s) XXX, XXX.*).<br>• If the data are all contained **within the manuscript and/or Supporting Information files**, enter the following: *All relevant data are within the manuscript and its Supporting Information files.*<br>• If neither of these applies but you are able to provide **details of access elsewhere**, with or without limitations, please do so. For example:<br><br>*Data cannot be shared publicly because of [XXX]. Data are available from the XXX Institutional Data Access / Ethics Committee (contact via XXX) for researchers who meet the criteria for access to confidential data.*<br><br>*The data underlying the results presented in the study are available*	All relevant data are within the manuscript and its Supporting Information files.

from (include the name of the third party and contact information or URL).<br>• This text is appropriate if the data are owned by a third party and authors do not have permission to share the data.<br><br>* typeset	
Additional data availability information:	

Powered by Editorial Manager® and ProduXion Manager® from Aries Systems Corporation

Manuscript Click here to access/download;Manuscript;Manuscript.docx

Cross-Sectional Study on Public Health Knowledge among First-Year University Students in Japan: Implications for Educators and Educational Institutions

Miwa Sekine[1,2*], David Aune[1], Shuko Nojiri[2], Makino Watanabe[3,1], Yuko Nakanishi[4], Shinobu Sakurai[7], Tomomi Iwashimizu[8], Yasuaki Sakano[5], Tetsuya Takahashi[6], and Yuji Nishizaki[1,2]

[1]Division of Medical Education, Juntendo University Faculty of Medicine, Tokyo, Japan

[2]Medical Technology Innovation Center, Juntendo University Faculty of Medicine, Tokyo, Japan

[3]Department of Physiology, Juntendo University Faculty of Medicine, Tokyo, Japan

[4]Juntendo University School of Health and Sport Science, Chiba, Japan

[5]Department of Radiological Technology, Juntendo University Faculty of Health Science, Tokyo, Japan

[6]Department of Physical Therapy, Juntendo University Faculty of Health Science, Tokyo, Japan

[7]Department of Public Health Nursing, Juntendo University, Faculty of Healthcare and Nursing, Chiba, Japan

[8]Juntendo University School of Health Sciences and Nursing, Shizuoka, Japan

* Corresponding author:

Email: miwa@juntendo.ac.jp (MS)

Short Title: Public Health Knowledge among First-Year University Students in Japan

Abstract

In recent years, health knowledge gaps and/or biases in health knowledge have become increasingly prevalent. These phenomena have become particularly noticeable and problematic during the COVID-19 pandemic and have further widened the spread of misinformation and knowledge disparities. With constant exposure to disinformation and misinformation through television, the internet, and social media, the lack of accurate public health knowledge is present even among healthcare-related university students. This study aimed to examine university students' knowledge on basic public health topics prior to admission to assess whether they had adequate knowledge before learning about further specialized education. Participants in this cross-sectional study were first-year students from medical schools, health-related colleges, and liberal arts colleges. A self-administered electronic survey was conducted from April to May 2021 at a private university in Japan, comprising six colleges with seven programs. Data analysis was conducted from June to December 2022 using students' self-reported public health knowledge, sources of information, and self-assessment of knowledge levels. Among the 1,562 students who received the questionnaire, 549 [192 male students (35%), 353 female students (64.3%), and 4 undisclosed (0.7%)] responded to at least one question. The results revealed that health knowledge, particularly sexual health knowledge, was limited, and at least 10% of students reported not learning in class before university admission the following 11 topics: two on Alcohol, Tobacco, and Other Drugs; eight on Growth, Development, and Sexual Health; and one on Personal and Community Health. These results indicated that there are significant knowledge gaps and biases as well as gender gaps concerning public health, especially sexual health, which may serve educators and educational institutions to better understand and prepare for further specialized education and suggest a need to supplement and reinforce the

foundation of public health knowledge in healthcare majors at the time of university admission.

Introduction

Public health knowledge is essential to a healthy society.

In recent years, the incidence of sexually transmitted diseases (STDs) has increased in industrialized countries [1-5], including Japan [6, 7], with a significant rise during the COVID-19 pandemic. The increase in STDs among females in their 20s and 30s, especially the prevalence of Chlamydia infections among females in their teens, is particularly notable in Japan [8, 9]. STD prevention is an integral part of public health, and it is taught in health and physical education in Japan. Historically, sexual education as an essential ingredient of health and physical education changed from “non-existent” to “teaching chastity” in 1949, after which sex-segregated sexual education was mainly concerned with physiological differences or merely teaching about menstruation, which further evolved to somewhat sex-integrated education in Japan only in recent years [10, 11]. In spite of the fact that topics such as gender equality, social justice, reproductive rights, and public health education have begun to be addressed, female students still prefer single-sex sexual education and female educators. In the presence of other sexes in the same room, sexual health can be uncomfortable to discuss or study [12], which makes conversations among them more challenging.

According to previous studies, there is a notable difference between the genders in their preferred sources of knowledge regarding sexual health. Female students’ ideal source of information was reported to be school as the primary source, followed by parents, friends, and the Internet. In contrast, male students preferred school as the primary source, followed by the Internet, friends, and parents, suggesting that there might be a difference in sexual education needs at home between male and female students [13]. Although diverse

information sources generally mean more accurate knowledge of sexual health [14], not all information sources provide accurate information [15, 16], especially information sources such as the internet. Internet-based information could potentially be harmful due to the presence of inaccurate, misleading, or incomplete information based on insufficient or unsubstantiated “scientific” evidence or even sensational, anecdotal views [16]. In fact, people with lower health literacy were more likely to use and trust health information from social media, blogs, or celebrity webpages [17].

Education regarding STD prevention and basic public health education is included in the standard curriculum guidelines issued by the Ministry of Education, Culture, Sports, Science, and Technology (MEXT) under Health and Physical Education in Japan [18, 19]. However, health and sexual health education are not necessarily required for enrollment in any college because health topics are not included in entrance exams, leaving the extent of public health topics to each educator’s discretion prior to university enrollment. This possible lack of public health education also applies to students enrolled in healthcare-related universities.

Healthcare professionals are required to disseminate this information. The lack of or inaccurate public health knowledge among healthcare professionals has a direct effect on the public. However, obtaining accurate health information is not necessarily equally easy for people of different social and economic backgrounds or even sexes. It has been reported that there are digital disparities, in which~~,~~ higher socio-economic status and higher education are factors positively associated with digital health information seeking, which could result from either a disparity of access~~,~~ or a disparity of literacy and comprehension [15, 16, 20]. There have also been gender gaps in education [21, 22]. In addition, there are other factors affecting public health knowledge, including promoting abstinence-only education, the lack of sexual health education altogether, or even the discouraging of its provision [23, 24] . With constant exposure to disinformation and misinformation through television, the Internet, and social

media [15, 25], it is imperative that younger generations, especially future healthcare providers, are given accurate health information that is the best available at the time.

Since the establishment of Health promotion in the 1986 Ottawa Charter by the World Health Organization in Ottawa, Canada [26], numerous studies, reports, and surveys have been conducted on sexual education worldwide. However, it is unknown whether future healthcare professionals will acquire adequate health and public health knowledge before enrolling in university.

In this study, we surveyed healthcare-related college students to assess their level of public health knowledge and evaluate whether they have adequate knowledge before proceeding to specialized education in healthcare-related universities. The findings of this study can be beneficial for educators to supplement the gap in students' essential knowledge, especially healthcare-related college students, in the aftermath of the COVID-19 pandemic.

Method

Study design and participants

We developed a self-administered questionnaire designed to assess and evaluate first-year university students' knowledge of public health topics and conducted a cross-sectional study at Juntendo University, Tokyo, Japan. In order to assess public health knowledge prior to university with minimal influence of health-related knowledge from university classes, we selected first-year students within two months of admission to Juntendo University. Juntendo University is a private university that focuses on health science, comprising six colleges with seven programs: medicine, health care and nursing, health science and nursing, health science/radiological technology, health science/physical therapy, health and sports science, and international liberal arts. Table 1 presents the program characteristics.

Table 1. Basic characteristics of Participants (n=549).

Colleges	Characteristics of colleges	Total (%)	Sex		
			Male (%)	Female (%)	Undisclosed (%)
**Medicine**	Private medical school established in 1838, headquarters in Tokyo	74(13.5)	37(50)	37(50)	0(0)
**Health Care and Nursing**	Nursing course serving greater Tokyo area	63(11.5)	1(1.6)	61(96.8)	1(1.6)
**Health Science and Nursing**	Nursing course serving in the local area community of Shizuoka prefecture	90(16.4)	6(6.7)	84(93.3)	0(0)
**Health Science/Physical Therapy**	Training course for physical therapists	83(15.1)	29(34.9)	54(65.1)	0(0)
**Health Science/ Radiological Technology**	Training course for radiological technologists	30(5.5)	9(30)	20(66.7)	1(3.3)
**Health and Sports Science**	Six courses related to sports. e.g., athlete, sports management, coaching, etc.	159(29)	94(59.1)	64(40.3)	1(0.6)
**International Liberal Arts**	Courses on global sociological issues, intercultural communication, global health service	50(9.1)	16(32)	33(66)	1(2)
**Total**		549(100)	192(35)	353(64.3)	4(0.7)

NOTE. Values in parenthesis represent percentage of valid total N.

We distributed web-based questionnaires to 1,562 first-year students at selected colleges using the university message system (Medicine: 136; Healthcare and Nursing: 204; Health Science and Nursing: 127; Health Science/Physical Therapy: 121; Health Science/Radiological Technology: 121; Health and Sports Science: 608; International Liberal Arts: 245) at Juntendo University from April to May 2021. We obtained informed consent from respondents by asking to tick the checkbox in the survey and collected the completed questionnaires. Respondents who did not provide informed consent were excluded from the study. Parental consent was not required since first-year students in Japan are 18 years of age or older. There were no other exclusion criteria.

Questionnaire and survey

To examine differences in students' awareness and knowledge of health and public health, we identified essential topics and health knowledge from the Japanese curriculum

guidelines, educational policies, educational contents of textbooks, and standard health education policies stipulated by the Centers for Disease Control and Prevention (CDC). We selected several crucial health education sections and topics shared by Japan and the United States: 1) Nutrition and Physical Activity; 2) Growth, Development, and Sexual Health; 3) Injury Prevention; 4) Alcohol, Tobacco, and Other Drugs; 5) Mental and Social Health; and 6) Personal and Community Health. We identified 31 knowledge topics for the educational content, which are listed in Supplemental Table S1. For each of these knowledge topics, we developed a set of three self-assessment questions, including: 1) the presence/absence of knowledge (acquired in class); 2) information sources for the knowledge; and 3) the level of knowledge on a 10-point scale ranging from 1 (no knowledge at all) to 10 (enough knowledge to teach someone else). In total, we prepared 86 self-assessment questions. We prepared five questions on knowledge of lifestyle-related diseases, contraception, LGBTQIA+ issues, pathogens causing infections, and transmission of infection, for a total of 91 questions (Supplemental Table S2). We developed the survey questions in consultation with medical and health education experts. We prepared a survey using Google Forms and administered it to all freshmen in all departments. All survey data was collected anonymously. The questionnaires included self-reported sex (i.e., male, female, undisclosed, or other). With a racially homogeneous environment and most university enrollment occurring immediately after high school, we did not ask about ethnicity or age to maintain anonymity. We consulted with two native English bilinguals and two native Japanese bilinguals to translate the questions and answers.

Data analysis

Descriptive analyses were performed to examine the respondents' characteristics and responses. Frequencies and percentages were utilized for categorical variables, and means

and standard deviations, or medians and 95% confidence intervals were utilized for continuous variables. If there was at least one answer to a question, the answer was accepted. Unless otherwise stated, the percentage was calculated based on the number of responses to each question. The number of topics for which students reported having knowledge and self-assessment knowledge levels was calculated as a percentage of the total knowledge score and total knowledge self-assessment score, and normality was tested using the Shapiro-Wilk test. We evaluated the total knowledge and self-assessment scores using Kruskal-Wallis tests and one-way analysis of variance, respectively, to evaluate whether there was a difference between colleges. The undisclosed sex group was excluded from the examination based on sex comparison. The Pearson's chi-square test was used to examine sex-based differences. Z-tests were used to further explore the differences, and the Bonferroni correction was employed to adjust for multiple comparisons. Mann-Whitney U tests were used to examine the mean rank differences in self-assessment by sex.

All statistical analyses were performed using IBM SPSS Statistics for Windows, Version 28.0 (Armonk, NY: IBM Corp.). The p-value threshold for significance was adjusted for multiple comparisons using the Bonferroni correction. The threshold for statistical significance was set at $p < .05$.

Ethics statement

This study was approved by the Juntendo University Ethical Review Board (No. 2020324) and followed the Strengthening the Reporting of Observational Studies in Epidemiology Reporting Guidelines. We obtained informed consent from respondents and collected the completed questionnaires.

Results

Response

Among the 1,562 students who received the questionnaires, 549 responded to at least one question (response rate: total 35.1%, medicine 54.4%, healthcare and nursing 30.9%, health science and nursing 70.9%, health science/physical therapy 68.6%, health science/radiological technology 24.8%, health and sports science 26.2%, and international liberal arts 20.4%). The study included 192 male (35%) and 353 female students (64.3%).

Knowledge of public health topics total score and self-assessment total score

Kruskal-Wallis tests shows that there were no significant differences among sexes [H(2)=3.763, *p*=.152] or among colleges in self-reported total knowledge scores in males [H(4)=3.813, *p*=.432], females [H(4)=8.175, *p*=.085], or total [H(4)=2.186, *p*=.702] (Table 2A). Additionally, the result of one-way analysis of variance shows that there were no significant differences between sexes [F(2, 494)=0.001, *p*=.999] or colleges on the self-assessment total score in males [F(4, 163)=0.353, *p*=.842], females [F(4, 320)=1.940, *p*=.104], or total [F(4, 492)=0.841, *p*=.500] (Table 2B).

Table 2. Summary of (A) students' reported public health knowledge score rate and (B) Self-assessment score.

	(A)Knowledge									(B)Self-assessment score					
	Male			Female			Total			Male		Female		Total	
	N	Median	95% CI	N	Median	95% CI	N	Median	95% CI	N	Mean±SD	N	Mean±SD	N	Mean±SD
**Medicine**	37	86.21	82.76-89.66	37	89.66	89.66-93.1	74	89.66	89.66-93.1	31	56.97±12.23	35	61.23±11.55	66	59.23±11.97
**Nursing**	7	89.66	86.21-100	145	86.21	86.21-89.66	153	86.21	86.21-89.66	7	61.53±11.13	132	56.01±13.39	140	56.16±13.34
**Health Care therapist**	38	87.93	86.21-89.66	74	89.66	89.66-93.1	113	89.66	89.66-93.1	34	57.35±9.85	67	54.84±12.51	102	55.78±11.67
**Health and sports**	94	84.48	79.31-89.66	64	89.66	86.21-96.55	159	86.21	86.21-89.66	82	55.68±15.21	58	58.44±11.5	141	56.83±13.77
**International Liberal Arts**	16	79.31	79.31-93.1	33	89.66	86.21-96.55	50	86.21	79.31-89.66	14	57.17±16.85	33	55.09±14.4	48	55.93±14.93
**Total**	192	86.21	86.21-89.66	353	89.66	89.66-93.1	549	86.21	86.21-89.66	168	56.63±13.66	325	56.67±12.89	497	56.66±13.12

NOTE: The number of topics about which students reported having knowledge and their self-assessment knowledge level for the topic were calculated to percentage as score. N represents the number of students who answered all questions about self-assessment. (S1 File. Questionnaire Raw Data.Sav)

Knowledge of public health topics and classroom involvement

There were 28 questions about whether students had knowledge of the topic and if they had learned it in class. More than 10% of the students were unfamiliar with 12 topics (two for Alcohol, Tobacco, and Other Drugs; nine for Growth, Development, and Sexual Health; and one for Personal and Community Health) of the 28 topics (Table 3A). More than 10% of the students responded that they did not learn about 11 topics (two for Alcohol, Tobacco, and Other Drugs; eight for Growth, Development, and Sexual Health; and one for Personal and Community Health) in class (Table 3B).

Table 3. Summary of students' previous knowledge regarding public health topics: (A) Did not know about the topic; (B) Did not learn about the topic in classes.

	**(A) Did not know about the topic**				**(B) Did not learn about it in classes**			
	**Total (N=545)**	**Sex (%)**		***p***	**Total (N=545)**	**Sex (%)**		***P***
		**Male (N=192)**	**Female (N=353)**			**Male (N=192)**	**Female (N=353)**	
Nutrition and Physical Activity								
**Lifestyle-related diseases**	46 (8.4)	19 (9.9)	27 (7.6)	.348	11 (2)	7 (3.6)	4 (1.1)	.044
**Food poisoning**	38 (7)	15 (7.8)	23 (6.5)	.570	21 (3.9)	10 (5.2)	11 (3.1)	.225
**Allergic reaction**	8 (1.5)	6 (3.1)	2 (0.6)	.018	7 (1.3)	5 (2.6)	2 (0.6)	.044
Alcohol, Tobacco, and Other Drugs								
**Health effect of smoking**	1 (0.2)	1 (0.5)	0 (0)	NA	0 (0)	0 (0)	0 (0)	NA
**Health hazard of passive smoking**	10 (1.8)	1 (0.5)	9 (2.5)	.092	5 (0.9)	1 (0.5)	4 (1.1)	.475
**Smoking effect on pregnancy**	31 (5.7)	15 (7.8)	16 (4.5)	.114	21 (3.9)	8 (4.2)	13 (3.7)	.779
**Dependence of smoking**	6 (1.1)	1 (0.5)	5 (1.4)	.341	2 (0.4)	0 (0)	2 (0.6)	
**Health effect of e-cigarettes**	197 (36.1)	63 (32.8)	134 (38)	.223	175 (32.1)	58 (30.2)	117 (33.1)	.470
**Effect of alcohol consumption**	9 (1.7)	4 (2.1)	5 (1.4)	.554	4 (0.7)	2 (1)	2 (0.6)	.531
**Alcohol consumption effect on pregnancy**	39 (7.2)	20 (10.4)	19 (5.4)	.030	24 (4.4)	11 (5.7)	13 (3.7)	.269
**Mental/physical effect of mind-altering drugs**	1 (0.2)	1 (0.5)	0 (0)	NA	1 (0.2)	1 (0.5)	0 (0)	NA
**Alcohol, smoking and drug addiction**	33 (6.1)	12 (6.3)	21 (5.9)	.888	17 (3.1)	7 (3.6)	10 (2.8)	.602

**Addiction**	117 (21.5)	45 (23.4)	72 (20.4)	.418	85 (15.6)	31 (16.1)	54 (15.3)	.805
Growth, Development and Sexual Health								
**Ovulation and menstruation**	17 (3.1)	15 (7.8)	2 (0.6)	<.001	8 (1.5)	7 (3.6)	1 (0.3)	.002
**Ejaculation**	21 (3.9)	1 (0.5)	20 (5.7)	.003	10 (1.8)	1 (0.5)	9 (2.5)	.094
**How to use condoms**	129 (23.7)	15 (7.8)	114 (32.3)	<.001	86 (15.8)	10 (5.2)	76 (21.5)	<.001
**Woman's basal body temperature**	190 (34.9)	103 (53.6)	87 (24.6)	<.001	139 (25.5)	76 (39.6)	63 (17.8)	<.001
**Measurement of woman's basal body temperature**	274 (50.3)	128 (66.7)	146 (41.4)	<.001	217 (39.8)	106 (55.2)	111 (31.4)	<.001
**Fertilization and pregnancy**	7 (1.3)	3 (1.6)	4 (1.1)	.666	2 (0.4)	1 (0.5)	1 (0.3)	.659
**Gestation period and childbirth**	67 (12.3)	30 (15.6)	37 (10.5)	.085	24 (4.4)	15 (7.8)	9 (2.5)	.004
**Public services related to pregnancy**	96 (17.6)	44 (22.9)	52 (14.7)	.015	63 (11.6)	32 (16.7)	31 (8.8)	.006
**Significance and importance of family planning**	155 (28.4)	60 (31.3)	95 (26.9)	.293	118 (21.7)	43 (22.4)	75 (21.2)	.768
**Abortion under Maternal Health Act in Japan**	98 (18)	46 (24)	52 (14.7)	.008	67 (12.3)	35 (18.2)	32 (9.1)	.002
**sex and gender**	131 (24)	33 (17.2)	98 (27.8)	.005	105 (19.3)	27 (14.1)	78 (22.1)	.022
**LGBTQIA+**	118 (21.7)	41 (21.4)	77 (21.8)	.862	96 (17.6)	33 (17.2)	63 (17.8)	.813
Personal and Community Health								
**Birth control pills and preventing sexually transmitted diseases**	206 (37.8)	73 (38)	133 (37.7)	.940	163 (29.9)	58 (30.2)	105 (29.7)	.913
**Prevention of sexually transmitted diseases**	28 (5.1)	12 (6.3)	16 (4.5)	.383	16 (2.9)	8 (4.2)	8 (2.3)	.208
**Vaccines**	26 (4.8)	11 (5.7)	15 (4.2)	.428	21 (3.9)	9 (4.7)	12 (3.4)	.445

NOTE: Values in parenthesis represent percentage of valid N for each category. Results are based on two-sided tests for comparison between sexes. The p-value threshold for significance was adjusted for multiple comparisons using the Bonferroni correction. The threshold for statistical significance was set at $p < .05$. (S1 File. Questionnaire Raw Data.Sav)

Knowledge of specific public health items

Questions on whether they had knowledge of specific items, diseases, or terms like lifestyle-related diseases, contraception, LGBTQIA+ issues, pathogens causing infections, and transmission of infection showed that more than 75% of students reported that they had knowledge of at least one or more specific items (Supplemental Table S3).

Information sources for public health topics

We inquired about the sources of information for 29 topics and found that most students relied on teachers as their primary source of information. However, for 12 out of 29 topics, less than 75% of students chose teachers as their source of information. For nine out of 29 topics, more than 25% of the students relied on their parent(s) for information. While friends were not necessarily the primary source of information, more than 10% of students obtained information on six public health topics from friends. Additionally, the Internet was an important information source for all the topics. However, for six out of the 29 topics, more than 40% of the students selected the Internet as their primary source of information, with more than 50% selecting the Internet for two of the topics (Table 4).

Table 4. Summary of source of information regarding public health topic.

	**Teacher(s) as information source**				**Parent(s) as information source**				**Friend(s) as information source**				**Internet as information source**			
	**Total (N=545)**	**Sex (%)**		***p***	**Total (N=545)**	**Sex (%)**		***p***	**Total (N=545)**	**Sex (%)**		***p***	**Total (N=545)**	**Sex (%)**		***p***
		**Male (N=192)**	**Female (N=353)**			**Male (N=192)**	**Female (N=353)**			**Male (N=192)**	**Female (N=353)**			**Male (N=192)**	**Female (N=353)**	
Nutrition and Physical Activity																
Lifestyle-related diseases	467 (85.7)	161 (83.9)	306 (86.7)	.397	142 (26.1)	58 (30.2)	84 (23.8)	.100	17 (3.1)	12 (6.3)	5 (1.4)	.002	208 (38.2)	83 (43.2)	125 (35.4)	.069
Food poisoning	332 (60.9)	114 (59.4)	218 (61.8)	.596	230 (42.2)	76 (39.6)	154 (43.6)	.366	25 (4.6)	10 (5.2)	15 (4.2)	.606	264 (48.4)	109 (56.8)	155 (43.9)	.004
Allergic reaction	362 (66.4)	121 (63)	241 (68.3)	.357	262 (48.1)	86 (44.8)	176 (49.9)	.362	61 (11.2)	24 (12.5)	37 (10.5)	.424	244 (44.8)	99 (51.6)	145 (41.1)	.010
Alcohol, Tobacco, and Other Drugs																
Health effect of smoking	491 (90.1)	174 (90.6)	317 (89.8)	.831	246 (45.1)	83 (43.2)	163 (46.2)	.491	45 (8.3)	24 (12.5)	21 (5.9)	.008	233 (42.8)	98 (51)	135 (38.2)	.004
Health hazard of passive smoking	481 (88.3)	166 (86.5)	315 (89.2)	.212	179 (32.8)	65 (33.9)	114 (32.3)	.761	33 (6.1)	26 (13.5)	7 (2)	<.001	187 (34.3)	80 (41.7)	107 (30.3)	.009
Smoking effect on pregnancy	423 (77.6)	152 (79.2)	271 (76.8)	.658	112 (20.6)	35 (18.2)	77 (21.8)	.293	14 (2.6)	9 (4.7)	5 (1.4)	.022	152 (27.9)	62 (32.3)	90 (25.5)	.105
Dependence of smoking	470 (86.2)	168 (87.5)	302 (85.6)	.530	186 (34.1)	72 (37.5)	114 (32.3)	.222	45 (8.3)	29 (15.1)	16 (4.5)	<.001	173 (31.7)	78 (40.6)	95 (26.9)	.001
Health effect of e-cigarettes	244 (44.8)	88 (45.8)	156 (44.2)	.685	103 (18.9)	39 (20.3)	64 (18.1)	.817	39 (7.2)	24 (12.5)	15 (4.2)	.001	166 (30.5)	74 (38.5)	92 (26.1)	.009
Effect of alcohol consumption	470 (86.2)	168 (87.5)	302 (85.6)	.637	207 (38)	78 (40.6)	129 (36.5)	.376	49 (9)	26 (13.5)	23 (6.5)	.007	182 (33.4)	75 (39.1)	107 (30.3)	.043
Alcohol consumption effect on pregnancy	435 (79.8)	157 (81.8)	278 (78.8)	.171	130 (23.9)	43 (22.4)	87 (24.6)	.636	19 (3.5)	10 (5.2)	9 (2.5)	.096	146 (26.8)	54 (28.1)	92 (26.1)	.512
Mental/physical effect of mind-altering drugs	501 (91.9)	173 (90.1)	328 (92.9)	.334	113 (20.7)	44 (22.9)	69 (19.5)	.338	32 (5.9)	17 (8.9)	15 (4.2)	.028	223 (40.9)	94 (49)	129 (36.5)	.004
Alcohol, smoking and drug addiction	480 (88.1)	169 (88)	311 (88.1)	.926	130 (23.9)	61 (31.8)	69 (19.5)	.001	39 (7.2)	25 (13)	14 (4)	<.001	179 (32.8)	77 (40.1)	102 (28.9)	.008
Addiction	381 (69.9)	136 (70.8)	245 (69.4)	.705	75 (13.8)	30 (15.6)	45 (12.7)	.446	17 (3.1)	9 (4.7)	8 (2.3)	.141	136 (25)	54 (28.1)	82 (23.2)	.303
Growth, Development and Sexual Health																

Ovulation and menstruation	475 (87.2)	166 (86.5)	309 (87.5)	.675	222 (40.7)	23 (12)	199 (56.4)	<.001	58 (10.6)	20 (10.4)	38 (10.8)	.980	180 (33)	46 (24)	134 (38)	.002
Ejaculation	453 (83.1)	159 (82.8)	294 (83.3)	.495	28 (5.1)	12 (6.3)	16 (4.5)	.417	88 (16.1)	52 (27.1)	36 (10.2)	<.001	152 (27.9)	89 (46.4)	63 (17.8)	<.001
How to use condoms	329 (60.4)	130 (67.7)	199 (56.4)	.854	9 (1.7)	3 (1.6)	6 (1.7)	.714	100 (18.3)	60 (31.3)	40 (11.3)	<.001	167 (30.6)	94 (49)	73 (20.7)	<.001
Woman's basal body temperature	273 (50.1)	82 (42.7)	191 (54.1)	.611	70 (12.8)	7 (3.6)	63 (17.8)	<.001	13 (2.4)	12 (6.3)	1 (0.3)	<.001	102 (18.7)	32 (16.7)	70 (19.8)	.901
Fertilization and pregnancy	488 (89.5)	172 (89.6)	316 (89.5)	.794	44 (8.1)	11 (5.7)	33 (9.3)	.131	46 (8.4)	28 (14.6)	18 (5.1)	<.001	141 (25.9)	67 (34.9)	74 (21)	<.001
Gestation period and childbirth	460 (84.4)	162 (84.4)	298 (84.4)	.760	93 (17.1)	18 (9.4)	75 (21.2)	.001	19 (3.5)	11 (5.7)	8 (2.3)	.033	127 (23.3)	50 (26)	77 (21.8)	.233
Public services related to pregnancy	351 (64.4)	121 (63)	230 (65.2)	.843	193 (35.4)	54 (28.1)	139 (39.4)	.020	7 (1.3)	4 (2.1)	3 (0.8)	.198	105 (19.3)	43 (22.4)	62 (17.6)	.101
Significance and importance of family planning	331 (60.7)	121 (63)	210 (59.5)	.193	90 (16.5)	31 (16.1)	59 (16.7)	.931	10 (1.8)	7 (3.6)	3 (0.8)	.018	93 (17.1)	40 (20.8)	53 (15)	.063
Contraception	427 (78.3)	154 (80.2)	273 (77.3)	.548	35 (6.4)	10 (5.2)	25 (7.1)	.381	90 (16.5)	48 (25)	42 (11.9)	<.001	215 (39.4)	99 (51.6)	116 (32.9)	<.001
Abortion under Maternal Health Act in Japan	386 (70.8)	135 (70.3)	251 (71.1)	.751	28 (5.1)	3 (1.6)	25 (7.1)	.006	8 (1.5)	4 (2.1)	4 (1.1)	.356	132 (24.2)	48 (25)	84 (23.8)	.622
sex and gender	343 (62.9)	135 (70.3)	208 (58.9)	.085	14 (2.6)	5 (2.6)	9 (2.5)	.918	50 (9.2)	24 (12.5)	26 (7.4)	.089	168 (30.8)	72 (37.5)	96 (27.2)	.051
LGBTQIA+	299 (54.9)	115 (59.9)	184 (52.1)	.038	31 (5.7)	12 (6.3)	19 (5.4)	.645	72 (13.2)	24 (12.5)	48 (13.6)	.763	277 (50.8)	102 (53.1)	175 (49.6)	.308
Personal and Community Health																
Prevention of sexually transmitted diseases	459 (84.2)	167 (87)	292 (82.7)	.081	31 (5.7)	9 (4.7)	22 (6.2)	.475	37 (6.8)	26 (13.5)	11 (3.1)	<.001	167 (30.6)	79 (41.1)	88 (24.9)	<.001
Pathogens that cause infections	430 (78.9)	152 (79.2)	278 (78.8)	.526	55 (10.1)	18 (9.4)	37 (10.5)	.745	22 (4)	14 (7.3)	8 (2.3)	.004	197 (36.1)	82 (42.7)	115 (32.6)	.010
Transmission of infection	409 (75)	151 (78.6)	258 (73.1)	.110	98 (18)	30 (15.6)	68 (19.3)	.307	25 (4.6)	19 (9.9)	6 (1.7)	<.001	286 (52.5)	107 (55.7)	179 (50.7)	.225
Vaccines	438 (80.4)	151 (78.6)	287 (81.3)	.713	133 (24.4)	45 (23.4)	88 (24.9)	.785	21 (3.9)	16 (8.3)	5 (1.4)	<.001	213 (39.1)	90 (46.9)	123 (34.8)	.003

NOTE: Values in parenthesis represent percentage of valid N for each category. Results are based on two-sided tests for comparison between sex. The p-value threshold for significance was adjusted for multiple comparisons using the Bonferroni correction. The threshold for statistical significance was set at $p < .05$. (S1 File. Questionnaire Raw Data.Sav)

Self-assessment of knowledge level

The lowest level of self-assessed knowledge was 4.0 (95% CI, 4.0–5.0) for topics such as the health effects of e-cigarettes, women's basal body temperature, and LGBTQIA+ issues. Meanwhile, the highest level of self-assessed knowledge was 7.0 (95% CI, 7.0–8.0) for topics such as the health effects of smoking, the health hazards of passive smoking, smoking dependence, the mental and physical effects of mind-altering drugs, and ovulation and menstruation.

Comparison of previous knowledge and class involvement by sex

There was a significant difference between males and females in their previous knowledge of topics such as allergic reactions, alcohol consumption effects on pregnancy, ovulation and menstruation, ejaculation, how to use condoms, women's basal body temperature, measurement of women's basal body temperature, public services related to pregnancy, abortion under the Maternal Health Act in Japan, and sex and gender (Table 3A). Moreover, there were significant differences between males and females in reporting that they did not learn about certain topics in their classes, such as lifestyle-related disease, allergic reactions, ovulation and menstruation, how to use condoms, women's basal body temperature, measurement of women's basal body temperature, abortion under the Maternal Health Act in Japan, and sex and gender (Table 3B).

Comparison of sources of information by sex

Significantly more male students selected their teacher(s) as sources of information about LGBTQA+. However, significantly more female students selected their parent(s) as the source of information on the six topics. Furthermore, significantly more male students

selected their friend(s) as a source of information than female students did for 20 out of the 29 topics. Additionally, significantly more male students reported the Internet as a source of information than female students for 16 out of 29 topics, while more female students reported the Internet as a source on one topic (Table 4).

Comparison of self-assessment by sex

The results of the Mann-Whitney U test demonstrated that male students reported significantly higher self-assessment scores on seven out of 29 topics, while female students reported significantly higher scores on five out of 29 topics (Table 5).

Table 5. Self-assessment of knowledge score comparison between sexes.

Key words	**Sex**			**Male**			**Female**			**p**
Nutrition and Physical Activity	**Mann-Whitney U**	**Wilcoxon W**	***Z***	**N**	**Mean Rank**	**Rank Sum**	**N**	**Mean Rank**	**Rank Sum**	
Lifestyle-related diseases	34776	96904	.786	190	264.5	50249	352	275.3	96904	.432
Food poisoning	34866	97347	.566	192	267.9	51439	353	275.8	97347	.571
Allergic reaction	38638	100414	2.984	191	244.7	46739	351	286.1	100414	.003
Alcohol, Tobacco, and Other Drugs										
Health effect of smoking	31622	94103	-1.314	192	284.8	54682	353	266.6	94103	.189
Health hazard of passive smoking	31031	93512	-1.653	192	287.9	55274	353	264.9	93512	.098
Smoking effect on pregnancy	38054	99830	2.525	192	249.3	47867	351	284.4	99830	.012
Dependence of smoking	29867	91995	-2.182	191	291.6	55701	352	261.4	91995	.029
Health effect of e-cigarettes	31109	93237	-1.449	191	285.1	54459	352	264.9	93237	.147
Effect of alcohol consumption	34666	97147	.555	191	267.5	51093	353	275.2	97147	.579
Alcohol consumption effect on pregnancy	35638	97414	1.339	190	258.9	49197	351	277.5	97414	.181
Mental/physical effect of mind-altering drugs	33824	95952	.019	192	272.3	52288	352	272.6	95952	.985
Alcohol, smoking and drug addiction	33391	95872	-.287	192	275.6	52913	353	271.6	95872	.774
Addiction	32058	92436	-.534	190	273.8	52017	347	266.4	92436	.593

Growth, Development and Sexual Health										
Ovulation and menstruation	56749	119230	13.155	192	153.9	29556	353	337.8	119230	<.001
Ejaculation	16419	78900	-10.037	192	364.0	69886	353	223.5	78900	<.001
How to use condoms	16618	77344	-9.668	191	357.0	68186	348	222.3	77344	<.001
Measurement of woman’s basal body temperature	46965	108741	8.176	188	195.7	36790	351	309.8	108741	<.001
Fertilization and pregnancy	30672	92800	-1.611	190	286.1	54353	352	263.6	92800	.107
Pregnancy and childbirth	35016	97497	.860	190	264.2	50199	353	276.2	97497	.390
Public services related to pregnancy	36979	98755	2.008	191	253.4	48398	351	281.4	98755	.045
Significance and importance of family planning	35190	95568	1.306	190	257.3	48886	347	275.4	95568	.192
Contraception	27879	89655	-3.189	190	299.8	56957	351	255.4	89655	.001
Abortion under Maternal Health Act in Japan	36010	97085	1.669	190	255.0	48446	349	278.2	97085	.095
sex and gender	29588	90663	-2.179	191	290.1	55408	349	259.8	90663	.029
LGBTQIA+	32848	93923	-.078	189	270.2	51068	349	269.1	93923	.938
Personal and Community Health										
Prevention of sexually transmitted diseases	27177	88953	-3.597	190	303.5	57658	351	253.4	88953	<.001
Pathogens that cause infections	27178	88603	-3.467	189	301.2	56927	350	253.2	88603	<.001
Transmission of infection	30853	92629	-1.362	189	282.8	53441	351	263.9	92629	.173
Vaccines	33252	93630	.376	188	264.6	49750	347	269.8	93630	.707

NOTE: Mann-Whitney U test was used for comparison. The p-value threshold for significance was adjusted for multiple comparisons using the Bonferroni correction. The threshold for statistical significance was set at $p < .05$. (S1 File. Questionnaire Raw Data.Sav)

Discussion

This study aimed to assess the knowledge, sources of information, and self-assessment of essential public health topics among university students before beginning their healthcare education, as well as to evaluate differences in the choice of colleges that indicate their desired career path. To the best of our knowledge, there has been no prior investigation

into the public health knowledge of healthcare university students before beginning their college education.

Although students reported being familiar of most of the health topics surveyed, there appeared to be a lack of knowledge in certain areas and reliance on skewed information sources, as detailed below.

Nutrition and Physical Activity

Ninety percent or more of the students affirmed their awareness of all three questions related to food poisoning and allergies, and reported their parent(s) and the Internet as sources of information. This finding suggests that education on health knowledge related to daily life is likely to be provided at home. Although many students reported the internet as their source of information, it is important to note that the information available on the internet is not necessarily accurate. This could be particularly critical in situations related to food poisoning or allergies, where inaccurate information could have serious consequences.

Alcohol, Tobacco, and Other Drugs

Numerous students reported obtaining knowledge about alcohol, tobacco, and other drugs both inside and outside the classroom. The information sources included teachers, parents, and the Internet. However, many students were unaware of the health impact of e-cigarettes and addiction as a disease. They stated that they had never learned about these two topics in school. Although e-cigarettes are relatively new, their negative health effects have been reported in Japan and worldwide [27, 28]. The regulations for conventional cigarettes do not apply to e-cigarettes [29], leading to many advertisements targeting younger generations that present e-cigarettes as a less harmful alternative. We believe that education on this topic is essential in schools.

Growth, Development, and Sexual Health

Nine out of the 12 topics had 10% or more students who reported having no knowledge, and 10% or more students reported that they had not learned about eight of these topics in class. In particular, fewer female students reported knowing how to use condoms, and fewer male students reported knowing about women's basal temperatures. Furthermore, male students reported the Internet as a source of information on how to use condoms, and female students reported parents as a source of information on the basal temperature of women. In addition, many students reported not having learned about the public services available during pregnancy, the importance of family planning, biological and social concepts of sex, gender identity, and what LGBTQIA+ stands for in school. They reported friends as the information source for these topics. Despite LGBTQIA+ individuals having specific healthcare requirements, curricula do not address these requirements in depth [30]. It is important for future healthcare workers to receive adequate education regarding sexual health and related public services, as well as LGBTQIA+-related health information, to provide appropriate healthcare to diverse societies.

Personal and community health

Interestingly, many students reported knowing about infection pathways, vaccination, and essential information during the COVID-19 pandemic, with the Internet having a high percentage as a source of information. It is speculated that this awareness may be due to the COVID-19 pandemic. This finding is consistent with previous reports on knowledge and information sources regarding COVID-19 from around April 2020 in nursing [31], medicine [32], medical and health science [33], and undergraduate and postgraduate students [34]. These previous reports confirmed that students had adequate knowledge of COVID-19, and for the majority of students, the source of information was social media. Furthermore, many

students reported that their sources of information about vaccination were their parents, compared with other topics. It can be speculated that vaccination, especially for minors, is a parental decision, even in the case of the COVID-19 vaccination. There have been reports that many people with vaccine hesitancy obtain information from the Internet and celebrities and tend to be suspicious of healthcare providers [35–38]. Therefore, education and communication are crucial [39, 40]. Environmental education studies on university freshmen have reported that the most important source of environmental education is the family, which provides long-term information and influences students' knowledge and attitudes [41]. Thus, although accurate knowledge must be imparted to children in school, parents should also have access to accurate sources of information.

Overview

This study revealed skewed information sources, with more male students relying on the Internet and friends as information sources and a disparity between sexes in knowledge and self-assessment, especially regarding sexual health. Our results are consistent with a previous study on the source of sexual knowledge, which reported that female students' ideal source of knowledge was school as a primary source, while male students preferred the Internet, friends, and parents in that order [13]. However, the disparity in class involvement for knowledge between the sexes on some sexual health topics in our study is notable. Although more female students reported that they did not learn about "how to use condoms" or "sex and gender" in class, more male students reported that they did not learn about topics related to women's sexual health, such as "women's basal body temperature" or "gestation period and childbirth". We could speculate about several possibilities to explain this disparity. One possibility is actual educational differences based on sex, while the other is perceived differences. Historically, sexual education in Japan was conducted separately based

on sex until recently [10, 42, 43]. Due to the long history of sexual education or lack thereof in Japan, students may consider topics not directly connected to their sex as irrelevant to them or not theirs to learn, thinking it is not their concern. With Japan ranking 120th out of 156 countries in 2021 in the Global Gender Gap Index, it is crucial to educate not only students but also parents and educators that sexual health education is equally essential regardless of sex. Teaching sex education as health education, rather than merely teaching physiological differences and reproductive functions, is crucial to raising awareness of infection prevention, especially sexually transmitted infections, as well as enriching students' lives to build a healthy society [44]. However, this survey also demonstrated that practical and essential knowledge of real life was somewhat limited, despite students' knowledge of physiological differences.

This lack of knowledge is likely contributing to the increasing rates of sexually transmitted infections among young people within Japan and worldwide [3, 5]. With the rise of HIV/AIDS in the 1980s [45, 46], the importance of health education, including sex education, came to the forefront. However, political and social background differences have resulted in varying approaches to sexual health education worldwide, with some countries promoting abstinence-only education, lacking sexual health education altogether, or even discouraging its provision.

During the COVID-19 pandemic, educational lectures and practical training were moved to online formats, including healthcare education [47, 48]. While some countries and regions have accelerated medical students' graduation and assigned them to work [49], the role of medical students during the pandemic remains controversial [50]. It is vital for the public, especially healthcare students, to have accurate public health information in this day and age. Thus, understanding these students' knowledge gaps provides an opportunity to supplement and reinforce their understanding of critical health topics.

Limitations

There are some limitations to this study. First, while we surveyed seven college programs, we only conducted the study at one Japanese university. Thus, we cannot ignore the possibility that the results would be different if the study were conducted at a different university or country. Second, to the best of our knowledge, we were unable to find a validated public health knowledge questionnaire suitable for this study, and hence, we selected topics from the curriculum guidelines and current official textbooks ourselves. To provide anonymity, obtain honest answers, and give students the opportunity to gain insight into their own information gaps, we did not test the students; instead, we had them self-report their knowledge or lack thereof. Thus, there is a possibility of inaccurate reporting of their knowledge. Thirdly, we cannot rule out the possibility that students who answer surveys are not representative of the entire population. Finally, this study was conducted during the COVID-19 pandemic. Although the study provides a glimpse into students' knowledge and attitudes in the age of the pandemic, the results may not be generalizable. Therefore, we recommend conducting further research on public health knowledge, both within and beyond Japan, to better understand the current state of health education and identify areas for improvement.

Implications

In Japan, health and sexual health education are not given much emphasis for enrollment in any college, including health-related colleges, as these topics are not included in entrance exams. Therefore, students seldom receive health education, let alone enough to proceed with higher healthcare education. Additionally, Japanese medical students' choice to attend medical school tends to be extrinsically rather than intrinsically motivated, meaning that they choose medicine because their grades are high enough for them to be admitted to

medical school. Although there were differences in knowledge on individual topics, there were no significant differences in total knowledge or total self-assessment scores between colleges. Although further research is needed, we can speculate that medical students are not necessarily focusing on learning each topic related to public health due to the reasons above, nor are they as interested in public health topics as can be expected. The health topics in this study are particularly crucial topics derived from preschool curricula and textbooks through the end of high school in Japan and the United States. Hence, they are not expected to be taught in universities or healthcare-related colleges. In other words, the lack of knowledge at this point may not be supplemented before students become healthcare workers. To address this issue, we believe that supplemental education reinforcing public health knowledge at the beginning of university education is beneficial. However, conventional classroom education may not be effective as students may not consider all topics to be of concern. Instead, we propose utilizing student-centered learning, such as problem-based learning [51], project-based learning [52], and flipped classrooms [53], as additional supplemental basic public health knowledge education after enrollment. We believe that this approach will lay the foundation for future healthcare workers' knowledge and, subsequently, promote the necessary knowledge and awareness indispensable for a healthy society.

Conclusions

In this cross-sectional study, we identified a significant knowledge gap and an overreliance on misinformation sources concerning public health, particularly sexual health, which varied by gender. This finding emphasizes the need for early public health education and awareness-raising efforts in universities, emphasizing the equal importance of health knowledge regardless of gender. Japanese education systems primarily focus on preparing students for university entrance exams, which has led to the neglect of health education as it

is not a mandatory part of the exam or university admission requirements. Given the widespread misinformation present in today’s world, a policy for improving health education is essential not only to address the lack of accurate knowledge and prevent healthcare workers from acquiring erroneous information but also to raise awareness and decrease the gender gap in sexual health. In conclusion, introducing healthcare-related knowledge to university students, particularly as part of freshmen or healthcare-related schools’ curricula, would be beneficial for building a healthy society. This approach would run in parallel with professional training and contribute to creating awareness about public health issues, ultimately leading to a better-informed population and a healthier society.

Acknowledgments

The authors express their gratitude to editage™ for their assistance in editing the manuscript. Additionally, we extend our thanks to the students who participated in this study.

References

1. Prevention CfDCa. Preliminary 2021 STD Surveillance Data 2022 [updated 2022-09-01T03:11:26Z. Available from: https://www.cdc.gov/std/statistics/2021/default.htm.
2. Serwin AB, Unemo M. Syphilis in females in Bialystok, Poland, 2000-2015. Przegl Epidemiol. 2016;70(2):273-80.
3. Stamm LV. Syphilis: Re-emergence of an old foe. Microb Cell. 2016;3(9):363-70.
4. Organization WH. Report on global sexually transmitted infection surveillance. World Health Organization; 2018.

5. Takahashi T, Arima Y, Yamagishi T, Nishiki S, Kanai M, Ishikane M, et al. Rapid Increase in Reports of Syphilis Associated With Men Who Have Sex With Women and Women Who Have Sex With Men, Japan, 2012 to 2016. Sex Transm Dis. 2018;45(3):139-43.

6. Ghaznavi C, Tanoue Y, Kawashima T, Eguchi A, Yoneoka D, Sakamoto H, et al. Recent changes in the reporting of STIs in Japan during the COVID-19 pandemic. Sex Transm Infect. 2022:sextrans-2021-0.

7. (NIID) TNIoID. Infectious Disease Weekly Report 2023 [Available from: https://www.niid.go.jp/niid/en/.

8. Hirohisa I. Chlamydia trachomatis infection among high school student(in Japanese). Japanese Journal of Chemotherapy. 2007;55(2):135-42.

9. Onodera S. Kinnen no wagakuni ni okeru seikansensho no doukou(in Japanese). Modern media. 2012.

10. Nishioka E. Historical transition of sexuality education in Japan and outline of reproductive health/rights. Nihon Eiseigaku Zasshi. 2018;73(2):178-84.

11. Satou T. A Study on the Meaning of Separated Learning and Integrated Learning between Male and Female Students in Sexuality Education in the Period of Puberty (in Japanese). Bulletin of the Faculty of Education, Mie University Natural science, humanities, social science, education. 2006;171:183.

12. ISHII R, KIYAMA K. A Study about Good Sex Education from the Student’s Viewpoint:Focused on University Students’ Learned Situation and Learning Needs (in Japanese). Annual report of the Faculty of Education, Gunma University Cultural science series. 2018;53:79-7.

13. Watanabe K, Tanaka H, Totsu Y. Sources of Sexual Knowledge for High School Students in Tokyo, Japan. Creative Education. 2018;09(15):2394-404.

14. Fisher CM, Kauer S, Singleton A, Walsh-Buhi E. An Examination of Sexual Health Information Sources, Knowledge and Behaviours Among Australian Teens: a Latent Class Analysis. Sexuality Research and Social Policy. 2022.

15. Wang Y, McKee M, Torbica A, Stuckler D. Systematic Literature Review on the Spread of Health-related Misinformation on Social Media. Soc Sci Med. 2019;240:112552.

16. Benigeri M. Shortcomings of health information on the Internet. Health Promot Int. 2003;18(4):381-6.

17. Chen X, Hay JL, Waters EA, Kiviniemi MT, Biddle C, Schofield E, et al. Health Literacy and Use and Trust in Health Information. Journal of Health Communication. 2018;23(8):724-34.

18. Ministry of Education C, Sports, Science and Technology. Section 7 Health and Physical Education 2008 [Available from: https://www.mext.go.jp/component/a_menu/education/micro_detail/__icsFiles/afieldfile/2011/04/11/1298356_8.pdf.

19. Ministry of Education C, Sports, Science and Technology. Health and Physical Education for High school (in Japanese) 2018 [Available from: https://www.google.com/url?sa=t&rct=j&q=&esrc=s&source=web&cd=&cad=rja&uact=8&ved=2ahUKEwi-7YbSro3-

AhWVEYgKHb2wCJwQFnoECCYQAQ&url=https%3A%2F%2Fwww.mext.go.jp%2Fa_menu%2Fshotou%2Fnew-cs%2F1417513.htm&usg=AOvVaw0BXiT8m9O8RtM55O5SVXBA.

20. Jacobs W, Amuta AO, Jeon KC. Health information seeking in the digital age: An analysis of health information seeking behavior among US adults. Cogent Social Sciences. 2017;3(1):1302785.

21. Evans DK, Akmal M, Jakiela P. Gender gaps in education: The long view¹. IZA Journal of Development and Migration. 2021;12(1).

22. Filmer D. The structure of social disparities in education: Gender and wealth. Available at SSRN 629118. 2000.

23. Kohler PK, Manhart LE, Lafferty WE. Abstinence-only and comprehensive sex education and the initiation of sexual activity and teen pregnancy. J Adolesc Health. 2008;42(4):344-51.

24. Santelli JS, Kantor LM, Grilo SA, Speizer IS, Lindberg LD, Heitel J, et al. Abstinence-Only-Until-Marriage: An Updated Review of U.S. Policies and Programs and Their Impact. J Adolesc Health. 2017;61(3):273-80.

25. Nguyen HT, Do BN, Pham KM, Kim GB, Dam HTB, Nguyen TT, et al. Fear of COVID-19 Scale-Associations of Its Scores with Health Literacy and Health-Related Behaviors among Medical Students. Int J Environ Res Public Health. 2020;17(11).

26. Organization WH. Ottawa Charter for Health Promotion 1986.

27. Sohal SS, Eapen MS, Naidu VGM, Sharma P. IQOS exposure impairs human airway cell homeostasis: direct comparison with traditional cigarette and e-cigarette. ERJ Open Research. 2019;5(1):00159-2018.

28. Nabavizadeh P, Liu J, Havel CM, Ibrahim S, Derakhshandeh R, Jacob Iii P, et al. Vascular endothelial function is impaired by aerosol from a single IQOS HeatStick to the same extent as by cigarette smoke. Tob Control. 2018;27(Suppl 1):s13-s9.

29. Grana R, Benowitz N, Glantz SA. E-Cigarettes. Circulation. 2014;129(19):1972-86.

30. Obedin-Maliver J, Goldsmith ES, Stewart L, White W, Tran E, Brenman S, et al. Lesbian, Gay, Bisexual, and Transgender–Related Content in Undergraduate Medical Education. JAMA. 2011;306(9).

31. Albaqawi HM, Alquwez N, Balay-odao E, Bajet JB, Alabdulaziz H, Alsolami F, et al. Nursing Students' Perceptions, Knowledge, and Preventive Behaviors Toward COVID-19: A Multi-University Study. Frontiers in Public Health. 2020;8.

32. Khasawneh AI, Abu Humeidan A, Alsulaiman JW, Bloukh S, Ramadan M, Al-Shatanawi TN, et al. Medical Students and COVID-19: Knowledge, Attitudes, and Precautionary Measures. A Descriptive Study From Jordan. Frontiers in Public Health. 2020;8.

33. Gohel KH, Patel PB, Shah PM, Patel JR, Pandit N, Raut A. Knowledge and perceptions about COVID-19 among the medical and allied health science students in India: An online cross-sectional survey. Clinical Epidemiology and Global Health. 2021;9:104-9.

34. Olaimat AN, Aolymat I, Shahbaz HM, Holley RA. Knowledge and Information Sources About COVID-19 Among University Students in Jordan: A Cross-Sectional Study. Frontiers in Public Health. 2020;8.

35. Freed GL, Clark SJ, Butchart AT, Singer DC, Davis MM. Sources and perceived credibility of vaccine-safety information for parents. Pediatrics. 2011;127 Suppl 1:S107-12.

36. Hilton S, Petticrew M, Hunt K. Parents' champions vs. vested interests: who do parents believe about MMR? A qualitative study. BMC Public Health. 2007;7(1):42.

37. Meleo-Erwin Z, Basch C, MacLean SA, Scheibner C, Cadorett V. "To each his own": Discussions of vaccine decision-making in top parenting blogs. Hum Vaccin Immunother. 2017;13(8):1895-901.

38. Jones AM, Omer SB, Bednarczyk RA, Halsey NA, Moulton LH, Salmon DA. Parents' source of vaccine information and impact on vaccine attitudes, beliefs, and nonmedical exemptions. Adv Prev Med. 2012;2012:932741.

39. Salmon DA, Moulton LH, Omer SB, DeHart MP, Stokley S, Halsey NA. Factors associated with refusal of childhood vaccines among parents of school-aged children: a case-control study. Arch Pediatr Adolesc Med. 2005;159(5):470-6.

40. Zimet GD, Rosberger Z, Fisher WA, Perez S, Stupiansky NW. Beliefs, behaviors and HPV vaccine: correcting the myths and the misinformation. Prev Med. 2013;57(5):414-8.

41. Maurer M, Bogner FX. How freshmen perceive Environmental Education (EE) and Education for Sustainable Development (ESD). PLoS One. 2019;14(1):e0208910.

42. Tsuyoshi ISKRS. Effect of Students Learning in Coeducational Physical Education Class. The bulletin of the Faculty of Education, Utsunomiya University Section 1 (in Japanese). 2020(70):277-89.

43. Ministry of Education C, Sports, Science and Technology. Health and Physical Education Curriculum Guideline 2019 [Available from: https://www.google.com/url?sa=t&rct=j&q=&esrc=s&source=web&cd=&ved=2ahUKEwj-gKbG7-79AhVytlYBHXAfB9YQFnoECBoQAQ&url=https%3A%2F%2Fwww.mext.go.jp%2Fcomponent%2Fa_menu%2Feducation%2Fmicro_detail%2F__icsFiles%2Fafieldfile%2F2019%2F03%2F18%2F1387018_008.pdf&usg=AOvVaw1nKTwBIBHabbTeraFbY5sg.

44. Goldfarb ES, Lieberman LD. Three Decades of Research: The Case for Comprehensive Sex Education. J Adolesc Health. 2021;68(1):13-27.

45. Curran JW, Jaffe HW, Centers for Disease C, Prevention. AIDS: the early years and CDC's response. MMWR Suppl. 2011;60(4):64-9.

46. Gallo RC. A reflection on HIV/AIDS research after 25 years. Retrovirology. 2006;3(1).

47. Alsoufi A, Alsuyihili A, Msherghi A, Elhadi A, Atiyah H, Ashini A, et al. Impact of the COVID-19 pandemic on medical education: Medical students' knowledge, attitudes, and practices regarding electronic learning. PLoS One. 2020;15(11):e0242905.

48. Sekine M, Watanabe M, Nojiri S, Suzuki T, Nishizaki Y, Tomiki Y, et al. Effects of COVID-19 on Japanese medical students' knowledge and attitudes toward e-learning in relation to performance on achievement tests. PLoS One. 2022;17(3):e0265356.

49. Harvey A. Covid-19: medical schools given powers to graduate final year students early to help NHS. BMJ. 2020:m1227.

50. Miller DG, Pierson L, Doernberg S. The Role of Medical Students During the COVID-19 Pandemic. Ann Intern Med. 2020;173(2):145-6.

51. Hmelo-Silver CE. Problem-based learning: What and how do students learn? Educ Psychol Rev. 2004;16:235-66.

52. Blumenfeld PC, Soloway E, Marx RW, Krajcik JS, Guzdial M, Palincsar A. Motivating project-based learning: Sustaining the doing, supporting the learning. Educ Psychol. 1991;26(3-4):369-98.

53. Akçayır G, Akçayır M. The flipped classroom: A review of its advantages and challenges. Computers & Education. 2018;126:334-45.

Supporting information

S1 Table. List of specific knowledge questions regarding public health topics and answer choices (multiple answers).

S2 Table. List of items in questionnaire and their corresponding key words in tables and figures.

S3 Table. Comparison of Students' previous knowledge between sexes.

S1 File. Questionnaire Raw Data.

Click here to access/download
Supporting Information
S1_Table.docx

Click here to access/download
Supporting Information
S2_Table.docx

Click here to access/download
Supporting Information
S3_Table.docx

Click here to access/download
Supporting Information
S1_File_Questionnaire Raw Data.sav
